# Applications and Trends of Machine Learning in Genomics and Phenomics for Next-Generation Breeding

**DOI:** 10.3390/plants9010034

**Published:** 2019-12-25

**Authors:** Salvatore Esposito, Domenico Carputo, Teodoro Cardi, Pasquale Tripodi

**Affiliations:** 1 CREA Research Centre for Vegetable and Ornamental Crops, 84098 Pontecagnano Faiano, Italy; salvatore.esposito01@gmail.com (S.E.); teodoro.cardi@crea.gov.it (T.C.); 2 Department of Agricultural Sciences, University of Naples Federico II, 80055 Portici, Italy; carputo@unina.it

**Keywords:** genotyping by sequencing, genome-wide association studies, QTLs dissection, genomics, nanopore, PacBio, phenomics, machine learning, microRNA

## Abstract

Crops are the major source of food supply and raw materials for the processing industry. A balance between crop production and food consumption is continually threatened by plant diseases and adverse environmental conditions. This leads to serious losses every year and results in food shortages, particularly in developing countries. Presently, cutting-edge technologies for genome sequencing and phenotyping of crops combined with progress in computational sciences are leading a revolution in plant breeding, boosting the identification of the genetic basis of traits at a precision never reached before. In this frame, machine learning (ML) plays a pivotal role in data-mining and analysis, providing relevant information for decision-making towards achieving breeding targets. To this end, we summarize the recent progress in next-generation sequencing and the role of phenotyping technologies in genomics-assisted breeding toward the exploitation of the natural variation and the identification of target genes. We also explore the application of ML in managing big data and predictive models, reporting a case study using microRNAs (miRNAs) to identify genes related to stress conditions.

## 1. Introduction

Current challenges in agriculture aim to safeguard the agricultural production by climate changes and emerging diseases to provide adequate food resources for the growing global population estimated to overcome nine billion in 2050 [1]. Yield trends are insufficient to reach this goal [2]. Therefore, it is imperative that researchers adopt novel strategies to accelerate crop breeding to significantly boost production. Next-generation sequencing (NGS) technologies, advanced phenotyping platforms, and machine-learning (ML) approaches are leading a new revolution in plant breeding. They facilitate the study of the genotype and its relationship with the phenotype, especially for complex traits, through mass sequencing of genomes and transcriptomes [3]. In the last decade, NGS has brought life sciences into the “big data era”, with an unprecedented growth of omics studies. Crop improvement has benefited greatly from genomics-assisted breeding, allowing the integration of genomics and phenomics in genome-wide association studies (GWAS) and facilitating the prediction of phenotype from genotype in genomic selection. These strategies have contributed tremendously to accelerating the development of new cultivars with desired characteristics [4]. However, considered alone, they may be insufficient to overcome limitations that may occur for the identification of gene function. The effectiveness of conventional approaches to detect rare variants and/or transcripts is limited, results being confined to the tissues examined and to different developmental stages. Despite the exponential increase in understanding the molecular dynamics of physiological mechanisms in plants, the exploitation of this knowledge by breeders has been an elusive goal, mainly due to the lack of translation of the biological information to a practical level. Presently, the rapid generation of complex big datasets offers a unique opportunity to use deep-learning approaches to investigate plant models. ML strategies play a central role in acquiring information from data produced from single or multiple experiments and/or shared by the scientific community. Indeed, they can be used as input for algorithms in the development and training of accurate predictive models. In this scenario, ML will speed the development of resilient crops identifying important associations that regulate a biological process.

In this review, we discuss the role of ML applied to crop breeding, highlighting the support given in the investigation of the molecular basis of agronomic and qualitative traits and further understanding of biological mechanisms. We first report recent advances in NGS and related applications, giving an outline of the benefits of sensing technologies and phenotyping platforms in agriculture. Then, we discuss how ML and related deep-learning algorithms can handle the large data obtained from NGS and phenotyping platforms for studies addressed to precision breeding, complex trait dissection, and gene discovery. Finally, we report a case study showing how ML can predict microRNAs (miRNAs) involved in the response to stress conditions in cold tolerant wild potato species *Solanum commersonii*.

## 2. Genomics Applied to Breeding: What We Gain from Short Reads

Recent advances in NGS technologies, computational analysis [5], quantitative genetics [6,7], genomics [8,9], phenomics [10,11] offer the opportunity to enhance crop selection programs through a multidisciplinary process based on data enrichment [12,13]. Array-based genotyping platforms and reduced representation-based methods such as genotyping by sequencing (GBS) or restriction-site associated DNA sequencing (RAD-seq) [14,15,16] drastically reduced the time and costs of analysis, paving a rapid way for genetic diversity studies, quantitative trait loci (QTLs) dissection and identification of genes underpinning traits of agricultural interest. In the present section we briefly outline results available in this field for Solanaceae, grains, and legumes, which represent the most important crops in terms of consumption and economic impact [17]. Our objective is not to review all recent findings but to focus on examples that demonstrate how this field is progressing.

In tomato, these technologies have been applied to get new insights on marker order and chromosomal recombination obtained through high-density linkage maps consisting of ~8800 array-based single nucleotide polymorphisms (SNPs) [16]. Similarly, Illumina.s Infinium and GoldenGate assay platforms were used in 40 lines to identify 7054 SNPs which revealed how the function of 200 genes was altered by defined substitution events [18]. More recently, GBS was used to construct a saturated genetic linkage map in a recombinant inbred line population derived from crossing the tomato breeding line NCEBR-1 with the wild tomato *S. pimpinellifolium* L. acc. LA2093 [19]. The authors proposed a new computational pipeline for the calculation of recombination breakpoints and genomic bins using over 140 thousand identified SNPs. Such approaches allowed a significant reduction of the interval region size in which previously identified QTLs of agronomic interest were located as well as the identification of the most probable underlying candidate genes. Furthermore, the strategy gave more precise positions of the two main fruit weight QTLs (*fw2.2* and *fw3.2*), decreasing the respective intervals by ten-fold. Similarly, a major QTL for lycopene content (*Lyc12.1*) was verified at high resolution, identifying *ζ-carotene isomerase* (*SlZISO*) as a causative gene. RAD-seq was instead used to identify a set of 24,330 SNPs in 99 *S. pimpinellifolium* germplasm accessions retrieved from the Tomato Genetic Resource Center (TGRC) genebank [20]. The authors showed the potentiality of this marker technology comparing previous studies with the 8K tomato SNP array. RAD-seq proved to be more appropriate to profile and define the genetic differentiation of *S. pimpinellifolium* accessions as well as to estimate linkage disequilibrium. Another targeted genotyping methodology used to investigate the diversity in tomato and eggplant is the Single Primer Enrichment Technology [21]. The method requires a priori information on target sequences for probe design. This approach is a valid alternative to random complexity reduction methods and arrays, being able to give better discrimination among domesticated and wild species with respect to previous methodologies and with a high degree of transferability between closely related species [21]. Moreover, it allows users to customize the panel of target markers.

In pepper, Illumina technology in combination with bulked segregant analysis allowed the identification of molecular markers tightly linked to potyvirus resistance 4 (*Pvr*4) [22]. Using this strategy, the syntenic regions between resistant and susceptible progenies were identified, and more than 5000 SNPs were converted into cleaved amplified polymorphic sequence (CAPS) markers and used to map *Pvr*4 locus in F_2_ mapping populations. In another study, GWAS based on ~750 thousands GBS-polymorphic sites facilitated the identification of four novel loci associated with pepper fruit shape and size [14].

In potato, GBS has been efficiently used for SNP discovery in accessions of different species (*Solanum tuberosum*, *S. phureja*, *S. cardiophyllum*, *S. sparsipilum* and *S. stenotonum*). Results were compared to the SolCAP 8K array [23], demonstrating comparable match rates between genotype calls (about 90%) and similar outcomes. Very recently, the improved Illumina 22K SNP array was used to perform a GWAS study for starch phosphorylation in cultivated potato [24], allowing identification of 14 SNPs in 8 diverse genomic regions.

Significant progress in marker-assisted genomics has also been made in legumes. Large-scale SNP data sets were developed by mapping multiple pools of GBS reads in accessions of the two major gene pools (Andean and Middle American) of common bean *Phaseolus vulgaris* [25]. This approach allowed the authors to identify over 200K SNPs in each gene pool which were used for GWAS of yield related traits in plants grown under both heat and drought stress environments. In chickpea (*Cicer arietinum* L.), GBS has been used to construct a high-density linkage map for the identification of QTLs associated with seed traits [26]. Putative candidate genes with seed specific expression profiles were identified in five robust QTLs. More recently, a major QTL for *Phytophthora* root rot resistance was identified in three different chickpea mapping populations developed from independent sources of resistance [27]. This allowed the fine mapping of resistance loci, providing a valid tool for assisted breeding and genomics in chickpea.

In wheat, genomic assisted breeding has used to establish mapping populations, such as bi-parental [28], multi-parent advanced generation inter-cross (MAGIC) [29,30,31,32] and core collections [33,34]. GWAS in those populations have been employed for the dissection of the genomic regions underlying main agronomic traits [31,33], and for the identification of resistance to stripe rust (*Puccinia striiformis* f. sp. *Tritici*) [34]. In rice, breakthroughs have been obtained in the development of “super-varieties” based on known elite alleles associated with grain yield and quality [35]. The authors demonstrate how the adoption of high throughput genotyping methodologies, leading to a well-designed marker-assisted selection (MAS) scheme, facilitates the selection of elite crop varieties.

The examples presented represent some of the studies where high throughput genotyping methodologies, have been successfully applied to gene mapping and discovery. In the short-term, however, these strategies will likely not offer a final and comprehensive solution, since they are conditional on first identifying mutations or modifications with large effect. By contrast, genomic selection (GS) based on ML algorithms promise to overcome the limitations of MAS for quantitative traits. Indeed, it determines the genetic potential of an individual instead of identifying specific QTLs. Using this strategy, GS models with moderately high prediction accuracies of 0.28–0.45 for grain yield and 0.45–0.62 for stem rust resistance of elite wheat breeding lines, were developed [36,37]. A limitation for GS is the high variability in prediction accuracies. To overcome this constraint, researchers in the field of crop genetics should make a greater effort in data sharing and integration and encourage the development of novel prediction models, ensuring accuracy [38]. For instance, Bernardo and colleagues [39] showed that using the whole set of markers available for genotyping, it is possible to achieve better prediction of breeding values than using subsets of markers found to be significantly associated with QTLs. This was also empirically confirmed by Heffner, et al. [40], who compared phenotypic selection, MAS, and GS prediction performance of 13 phenotypic traits in 374 winter wheat (*Triticum aestivum* L.) breeding lines.

In conclusion, advances in genomics knowledge and development of “ultra-high throughput” technologies are providing breeders new tools for gene discovery and genetic dissection of complex traits, providing extraordinary opportunities to accelerate selection programs and develop novel cultivars. In the future, an increase in availability of genomic information is expected for mapping populations and the thousands of accessions of wild and cultivated species. Therefore, the development of novel pipelines for data analysis and the enhancement of computational capacities are mandatory to allow ML to build robust algorithms able to make better predictions.

## 3. Third Era of Generation Sequencing: The Impact on Plant Genetics

With the advent of Nanopore and Pacific Bioscience technologies the third-generation sequencing era began [41,42]. They provide a cheaper and faster method for genome sequencing. Compared to other technologies, they produce longer reads exceeding several kilobases, solving problems for the resolution of the assembly and the presence of repetitive regions in complex genomes. Nanopore is based on threading a single-stranded DNA molecule electrophoretically through tiny bio-pores with nanoscale diameters (inner diameter of 1 nm). Nanopores are divided into biological and solid-state [43]. The former is made by proteins (similar to those of biological membranes) whereas the latter is generally made by synthetic materials such as silicon nitride, which gives better stability and allows multiplexing on a single device. Each nanopore has a polymerase positioned near the pore entrance that incorporates four modified nucleotides into the growing copy of the DNA template strand. Each of the four distinct tags partially blocks the channel when held within the barrel of the nanopore, creating a unique signal. By contrast, Pacific Biosciences uses the same fluorescent labelling as the other sequencing approaches, although it detects the signals in real time. The method uses small wells of a few nanometers in diameter known as zero-mode waveguides harboring a DNA polymerase and a fragment of the target DNA. During sequencing, a luminous signal recorded by sensors is released whenever a nucleotide is incorporated in the new template. The DNA sequence is determined by the detection of labelled nucleotides.

In comparison to other technologies, Pacific Bioscience and Nanopore have several advantages. The preparation of the sample is very fast, taking only a few hours instead of days required by other technologies. In addition, both methods produce long reads (~10 kb), helping researchers to annotate regions of the genome highly abundant in repeat sequences [44]. As a counterpart, a high error rate (~13%) in insertions and deletions sequencing occurs with both methods [45].

In bacteria, genome assemblies of 9 strains by Oxford Nanopore Technology (ONT) have been reported to be highly accurate, although lack in contiguity has been encountered [46]. By contrast, highly contiguous genome assemblies using Nanopore were reported for 15 *Drosophila* species [47], with an average of 29x depth-of-coverage data and a contig N50 of 4.4 Mb. Interestingly, the authors showed how the updated genomes could be used to close over 60% of the gaps present in the currently published reference genome. They also demonstrated the power and cost-effectiveness (approximately $1000 for materials and reagents required for each genome) of long-read sequencing for genome assembly. In human genetics, nanopore applications are becoming widely used in clinical medicine and diagnostic contexts to identify the treatment of choice. Recently, Bowden and collaborators [48] re-analyzed the reference sample “NA12878” and sequenced the genome of an individual with ataxia-pancytopenia syndrome and severe immune dysregulation. Although the error rate from Nanopore remains substantially higher than that from short-read methods, the authors demonstrated the substantial benefits of analytical innovation, identifying two new non-synonymous de novo variants. Moreover, long-read sequencing has been reported to be successfully applied to complete the first assembly of a human chromosome X [49].

Most publications that demonstrate early application of Nanopore and PacBio technologies have been related to bacteria, virus, and human studies; however, the benefits of these sequencing technologies are now also being realized in the sequencing of plant genomes. Very recently, PacBio technologies were used to create genome assemblies of two cultivated allotetraploid cottons, *Gossypium hirsutum*, and *G. barbadense* has been released using PacBio technologies [50]. These new genome assemblies show significant improvements in the accuracy and completeness for regions with high content of repeated sequences (e.g., centromeres). Similarly, a de novo assembly approach was applied to reveal the lettuce genome sequence through Nanopore. This produced 1169 contigs, with N50 size of 7.3 Mb for a total of 2.6 Gb, representing nearly the entire lettuce genome. Furthermore, the combination of the obtained nanopore data with optical mapping allowed a better resolution, generating a scaffold with N50 of 146 Mb with chromosome-level assembly consisting of 34 scaffolds [51].

Further improvements have been developed to link sequence reads with assembly. Technologies such as linked-reads sequencing (10x Genomics) provides a new platform integrating Illumina sequencing with a microfluidic-based barcoding strategy. The technology combines large DNA fragments with barcoded gel beads. Libraries of reads with the same barcodes are then developed and sequenced [52]. The main advantages of linked-reads rely on a better identification of genome structural variants. The combination of several sequencing methodologies was applied during genome sequencing of Komodo dragon [53], where 10x Genomics linked-read sequencing, Bionano optical mapping data, PacBio sequencing and Oxford Nanopore MinIon sequencing provided sufficient resolution to give novel insights into understanding the biology of adaptation and the evolutionary signatures of this reptile.

Whole-genome re-sequencing is expected to be cost-affordable in the next decades, making this methodology accessible to a growing number of plant geneticists and breeders. However to make best use of these technologies for crop improvement the main targets for the near future are: development of suitable advanced genetic materials to dissect at a genomic and phenomic level as a source of novel traits for plant breeding; the improvement of computational pipelines for data processing and accessible analyses.

## 4. Machine Learning for Genomic Studies

The advent of third-generation sequencing technologies allows production of longer reads in comparison to the standard short reads obtained by Illumina sequencing (10–100 kbp instead of ~100 bp). However, a larger amount sequencing errors (5–15%) compared to Illumina (~1%) still occur, meaning SNP and small indel variant calling using both technologies remains a big challenge. To overcome this constraint, artificial neural networks are becoming prominent. Very recently, Luo and colleagues [54] released Clairvoyante, a convolutional neural network model to predict SNP or indel variants, zygosity, and indel length from aligned long reads. The authors tested their new approach on data produced with Illumina, PacBio, and ONT. In particular, they focused at the common variant sites with a minor allele frequency ≥ 5% from 1000 Genomes Project and evaluated Clairvoyante performance to call variants in a genome-wide scenario. The authors achieved 99.67%, 95.78% and 90.53% F1-score (a measure of test accuracy) when common variants were analyzed, and 98.65%, 92.57% and 87.26% in whole-genome analysis for Illumina, PacBio, and Oxford Nanopore data, respectively. Another application of artificial neural networks was given by Poplin and colleagues in the release of their the DeepVariant package [55]. For each variant site, DeepVariant computes the probabilities of three possible allele combinations (homozygous or heterozygous alleles with the reference, and homozygous alleles within the variants), by learning statistical relationships between images of reads around putative variant and true genotype calls. DeepVariant can learn using different sequencing technologies, including 10x Genomics and Ion Ampliseq exomes, highlighting the benefits of using more automated techniques for variant calling and allowing accurate prediction about the presence of a candidate variant. However, ML algorithms are not only limited to detect variants from long-read approaches but can be used by researchers working in the field of population genetics. Indeed, supervised ML has been applied to study the rates of recombination in a target genome. An example is given by Schrider [56], who used the random forest classifier to distinguish recombination rate classes in *Drosophila melanogaster* based on sequence motifs; the authors demonstrated that such motifs are predictive of recombination rate. Presently, many start-up companies are producing solutions to combine genomics and ML to release new tools aimed at predicting diseases in crops or to perform bioinformatic pipeline without coding knowledge. Examples of new technology providers include Trace Genomics and Sequentia Biotech. The former mainly focuses on applications aimed at soil health using proprietary ML models to identify the factors responsible for driving crop outcomes, whereas the latter has released tools such as artificial intelligence RNA-seq (AIR) as a solution to close the gap between data production and interpretation in transcriptomics [57]. Using AIR, researchers may obtain full differential gene expression reports within a few hours. The future perspective of ML will focus on how to deal with multiple different species simultaneously. Possibly deep-learning approaches might prove able to address comparative genomics analyses or the transfer of knowledge from a model plant to a crop of interest.

## 5. Machine Learning for Plant Phenomics and Smart Agriculture

**Figure 1:**
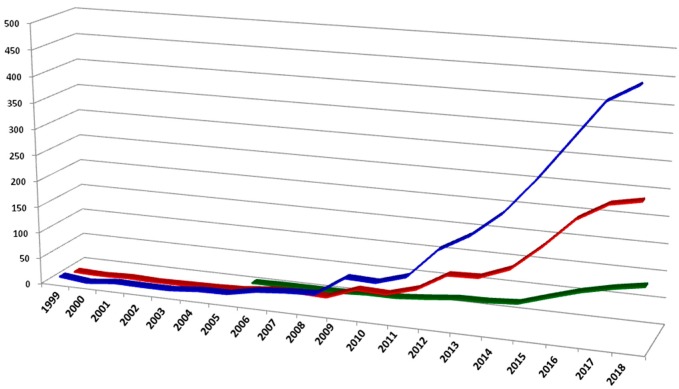
Number of indexed publications in the last 20 years concerning plant phenotyping (source Scopus). A search query in Title-Abstract-Keywords for: phenotyping and plant (blue line), phenotyping and crop (red line), phenotyping and sensors (green line).

Phenomics is a field of biology dealing with the deep characterization of qualitative and quantitative traits in plants. Collection and analysis of a wide range of measures and development of multivariate models able to dissect factors involved in the expression of the phenotype are the main pillars of this discipline. Next-generation phenomics combines precision in trait detection and big data generation by means of high throughput agri-systems and high-performance computing technologies. Conventional methods for plant traits analysis have performed relatively well for qualitative traits and mass selection; however, by comparison they have been slow to improve the effectiveness of understanding complex traits. Moreover, the phenotype of individuals is due to complex interactions between the genotype and environment, dissection of which requires precise determination of factors such as weather conditions, soil composition, and available water. Therefore, a better correlation between plant performance, environmental response, and gene function is possible through advanced phenotyping technologies. Sensing devices in agriculture provide a wide range of applications: from the control of indoor and outdoor cultivation conditions to the understanding of main physiological changes in plants because of external stress [58]. In the last decade, there has been a rapid increase of published research in the field of “sensing technologies and phenotyping of crops”, aiming to cover the gap with genomic science (Figure 1).

Indeed, the strong contribution given in the understanding of genes function from sequencing and re-sequencing projects of crop genomes is well known. Despite the plethora of information retrieved from this research, great efforts are still required to link plant phenotypes and genomic data and exploit those genes with unknown function. In this frame, ML plays a pivotal role for the analysis of complex agricultural data related to plant features and environmental parameters. It allows processing the huge amount of data from sensors and phenotyping platforms, increasing the throughput and accuracy in analysis, as well its management.

Plant phenomics relies on three main stages: (i) detection of a target trait such as a physiological process or a specific stress; (ii) extrapolation of data from devices (i.e., imaging analysis to quantitative measures or qualitative categories; (iii) computation, aiming to give a biological response from retrieved data and to support decision-making. The first step is mainly related to the technology used: HTTPs (high throughput phenotyping platforms, i.e., Lemnatec, Phenospex) or phenomic tools (i.e., spectrophotometer, fluorometer) overcome drawbacks occurring with conventional methods, allowing a rapid assessment and large-scale phenotyping, and ensuring precision, reproducibility, and accuracy in data acquisition helping to reduce bias. The second and third steps rely on computing approaches. ML algorithms help in the development of the best classification model for measuring and classifying traits (e.g., stress) as well for developing prediction models. Different ML approaches, divided in supervised and unsupervised, can be modified and applied for plant phenotyping. The supervised normalized cut (SNC) method incorporates training data and is used to detect, classify, and identify data from radiation detectors. Similarly, Support vector machine (SVM) is a supervised ML method which has been applied in humans to classify individuals in high-dimension space [59], face detection [60], and neuro-image classification [61]. By contrast, variance modeling at the observational level (Voom) method has been proposed to analyze transcriptomic data derived from RNA-Seq studies aimed at identifying relevant genes across contrasting conditions (e.g., disease and non- disease conditions) [62]. Successful examples of ML combined with prediction models applied to detect stresses in plants include the work of Goshal and collaborators [63], from the analysis of over 25,000 images of soybean leaflets subjected to various types of diseases and nutritional deficiencies, developed a convolutional neural network (CNN) able to dissect the image features at high resolution. A derived learning model for the identification, classification, and quantification of the applied stresses was developed. The authors proved the accuracy of the ML framework comparing its prediction with human expert ratings. A high level of agreement between the two types of diagnosis was found. Moreover, the transfer learning capability was investigated, showing how the CNN was suitable to detect the stress in other plant species. Another recent example reported a ML pipeline based on plant imaging segmentation [64]. The approach was based on the acquisition of many images of plant samples and subsequent processing using a random forest algorithm able to discern different parameters related to plant growth (e.g., leaf area). The combination of a high throughput hardware system with ML-based analysis allows the acquisition of real time images, convert them into numerical data, and predict the phenotype of interest. 

Despite the progress achieved in the field of ML, several concerns remain for plant phenotyping and genomics: (1) large dataset are required to develop a robust model, which is a time and cost consuming task; (2) ML models are not “universal”, needing further validation steps when switching to diverse crops; (3) the environment has the main influence on the variation of agricultural traits, therefore the transition from “in silico” to “real” may introduce incorrect estimations and possible bias; (4) the need for a highly qualified staff and high-tech equipment limit its use to the big companies and/or main research institutions.

## 6. Machine Learning for Next-Generation Breeding 

To fully exploit the advances of NGS technologies requires an understanding of structural and functional variations of genomes underpinning the changes in crop performance. To meet this goal, genomic data need to be integrated into appropriate models developed for the investigation of the complex interactions between crop performance and environmental factors. In this frame, ML methods play a key role in processing and association of the various sources of data, giving the possibility to develop accurate breeding programs. Breeding efforts to develop new varieties often involve multiple generations of crosses and selection, and large-scale trials to assess the performance of progeny. This process often requires several years and large numbers of individuals to test, due to the polygenic nature of most of the traits of agronomic interest. Predictive analysis implemented in ML gives a better chance to achieve results in a reasonable timeframe, providing breeders a road map based upon the different sources of information gathered across environments and years.

ML algorithms for precision breeding have been developed for the prediction of untested phenotypes in GS schemes or for the training of phenotypic variables using random forest algorithm [65]. The former method combines genomic markers in a training population [with phenotype data] to obtain genomic estimated breeding values (GEBVs) of individuals in a testing population that have been genotyped but not phenotyped [66]. By means of genotypic data it is then possible to perform the selection of unobserved individuals, reducing time and cost in variety development. Random forest is instead a method that uses multiple learning algorithms for regression and classification of variables to estimate the potentiality of predictors for decision-making. ML in GS can be performed through different parametric and non-parametric computational methods implemented in data-mining open-source software such as R [67]. Among the former, least absolute shrinkage and selection operator (LASSO), genomic best linear unbiased prediction (GBLUP) and Bayes models (A, B and C) are commonly used in plant breeding. Genotypic and phenotypic traits are not the only parameters to be considered in the selection. Environmental variance needs to be estimated due to the pressure given on those traits with medium-low heritability. GBLUP models can incorporate environmental covariates into genetic prediction and estimate the genotype by environment interaction, improving the accuracy of prediction for untested individuals in diverse locations. Among non-parametric methods, reproducing kernel Hilbert space (RKHS), SVM regression and artificial neural networks (ANN) are the most used prediction tools. A comparison of the various statistical GS methods on the basis of predicted accuracies in a simulated F_2_ progeny, revealed a higher accuracy of parametric approaches for populations with additive genetic architecture, while the epistatic interactions are better predicted by the non-parametric ones [67]. Therefore, multiple models can be applied by data analysts in supporting decision-making by plant breeders. In the last few years, on-line sources have been developed for the prediction of GEBVs. For example, solGS, a user-friendly on-line interface implemented in the Nextgen Cassava breeding database [68], which allows users to create training populations, input a dataset, and estimate the GEBV of selection candidates. The interactive on-line exploration and graphical data output makes this tool available to broad number of users.

## 7. Machine Learning and Big Data Management

**Figure 2:**
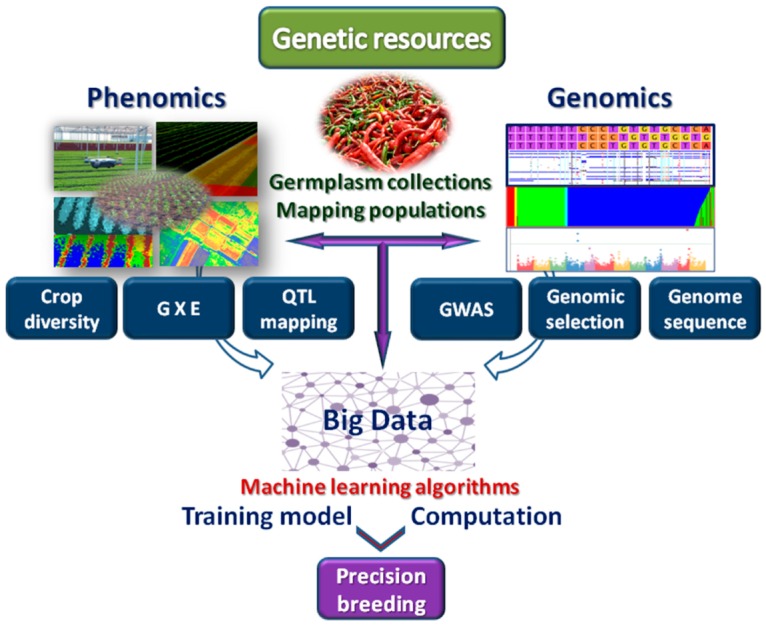
Integration of genomics and phenomics for the exploitation of genetic resources in genome wide association studies (GWAS), genotype by environment (GxE) estimation, quantitative trait loci (QTLs) analysis, investigation of crop diversity and genomic selection. Related big data are exploited in ML-based algorithms implemented in computational tools leading to precision breeding.

Handling NGS data requires high storage spaces and innovative computational capacities for the management of big data. To address this challenge, several repositories have been implemented in recent years. Raw data from sequencing projects are stored, together with their quality scores, in the Sequence Read Archive (SRA) [69], a repository adopted to store short sequence reads. This database stores raw reads from high throughput sequencing platforms, including Roche 454, Illumina, Applied Biosystems Sequencing by Oligonucleotide Ligation and Detection (SOLiD), Helicos Heliscope, Complete Genomics, and Pacific Biosciences single-molecule real-time sequencing (SMRT). However, data generated by NGS are rapidly growing (from hundreds of terabytes to petabytes in recent years) and their storage is becoming a major concern in data computing. Presently, only a few organizations, such as the European Bioinformatics Institute (EMBL-EBI) and the National Center for Biotechnology Information (NCBI), can store large datasets, providing free access to the scientific community. Companies such Amazon Simple Storage Service (Amazon S3) are emerging as alternative providers, offering a cloud-based file system, with virtually unlimited capacity. This system is being used as mirror of NCBI for the storage of a subset of human genomics data (about 200 terabytes) [70]. Other examples are Genewiz, a services company located in the USA, which provides similar services as Amazon S3, and Beijing Genomic Institute (BGI, Shenzhen) that has built a cloud-based data service for bioinformatics method development, automated analysis, and data delivery. Big data hosted in the cloud therefore represents a promising storage solution. Storage represents the preparatory step prior to the processing of data. A further phase is related to data analysis. Most of the NGS analysis software are command-line-based, leading to accessibility problems for many biologists and difficulty in selecting the best performing/most appropriate tools to use. In this frame, ML approaches, as a skill of computers to learn and process data without being a priori programmed, give the opportunity to better address accessibility aspects. Indeed, in addition to predictive analysis, ML can be implemented in tools able to execute and manage data automatically via algorithms for data categorization and cataloguing. Different source of data from large datasets to individual tables can be classified in multiple ways according to the requirements of users to enrich identifier driven searches and queries allowing richer cross-category analytics correlations. The potentialities of ML can be applied to give a better integration of data retrieved from genomics and phenomics, accelerating the discovery of trait-allele associations and developing appropriate models for precision breeding (Figure 2).

## 8. Genomics and Machine Learning: A Case Study to Predict Differentially Expressed miRNA

Among principal food crops, the cultivated potato (*Solanum tuberosum*) is the fourth largest in terms of production after corn, rice and wheat [17]. Exposure to several abiotic stresses threaten cultivations and production. Among them, low temperature is the main stressor, leading breeders to seek new sources of genetic resistance. Among the over 200 wild potato species commonly distributed in the Centre of origin and surrounding areas, *S. commersonii* displays high tolerance to low temperatures being able to acclimatize to the cold (i.e., to increase its resistance to cold following exposure to low but non-killing temperatures). The *S. commersonii* cold tolerant phenotype has attracted substantial research interest, having been studied using both classical breeding as well as omics approaches [71,72,73,74]. Furthermore, it has been the first wild potato species with a fully elucidated genome sequence [75]. All these studies provided a significant resource for clarifying in detail the molecular dynamics underlying physiological mechanisms of tolerance to low temperatures. They also suggested a possible role of microRNAs on the adaptative responses of this species to cold stress.

**Figure 3:**
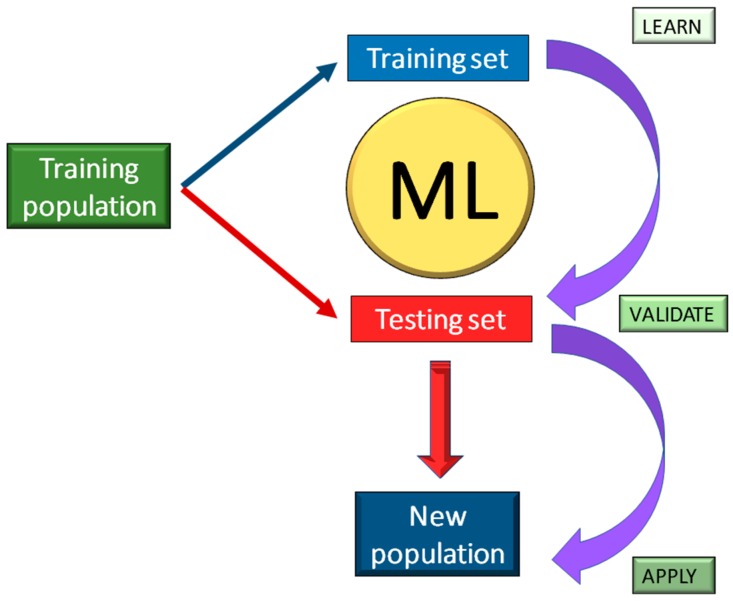
Flow chart of a Machine-Learning (ML) approach. A training population in divided in a training set (where ML makes prediction) and in a testing set (where ML validates the results and its accuracy is estimated). The validated model may now be applied to a new population.

MicroRNAs (miRNA) are small and highly conserved non-coding RNA molecules, encoded by *Dicer* genes (DCL) and involved in the regulation of gene expression [76,77]. Since the first discovery of miRNAs, the basic understanding of how gene expression is controlled deeply changed. In the last ten years, studies demonstrated that miRNAs play crucial roles in modulating stress response, including salinity [78], drought [79], waterlogging [80], UV-B radiation and cold stress [81,82], and in several cases their biological role has been clarified. For instance, miR394 and miR397 have been reported to positively regulate cold tolerance via the CBF-dependent pathway [83,84], whereas miR319 activates the CBF regulon and triggers reactive oxygen species (ROS) elimination from the cells [85]. Similarly, miR396 and mi408 have been demonstrated to act as positive regulators of cold tolerance through the modification of cellular antioxidant capacity [86,87]. Nevertheless, it seems that the ability of biological approaches to identify miRNA related to stress conditions depends on the relative frequency of transcripts, the tissues examined, and the developmental stage of the organism. Although quality controls have been applied during sample preparation and data analysis, there are still limitations on miRNA detectability, related to the alignment of reads, the normalization of data, and the applied statistical methods. As a result, the accuracy and sensitivity of the analysis can be influenced by false-positive (type I error) or false-negative (type II error) results. These constraints have led to the development of sophisticated ML approaches attempting to identify possible genes and miRNAs. In the present report, we tested seven different ML approaches (SVM, SNC, PLDA, PLDA2, NBLDA, VoomNSC, VoomDQDA) on published miRNAs data related to cold tolerance in *S. commersonii*. In a previous study, two clones of *S. commersonii* contrasting in their cold response were analyzed through small non-coding RNA sequencing (sncRNAome), revealing a possible role of sncRNA in the regulatory networks associated with tolerance to low temperatures and providing useful information for a more strategic use of genomic resources in potato breeding [71,72]. Samples were randomized without any classification and a learning model was applied to the raw data sets to make predictions on the susceptibility and the tolerance on a new developed sample set (Figure 3). 

By means of this strategy, 100% of samples were correctly classified in either tolerant or susceptible genotypes and 52 miRNAs were predicted as main features associated with cold stress tolerance. Twenty-seven miRNAs were in common with the previous study [71], whereas 25 were predicted as related to cold tolerance only by MLseq approaches.

### 8.1. Materials and Methods

Row counts of predicted miRNAs in 36 samples relative to two clones of *S. commersonii* contrasting in their ability to withstand cold stress (clone cmm1T cold tolerant and clone cmm6-6 cold susceptible) were used as input for MLseq [63]. First, samples were grouped as follows: 18 samples belonging to clone cmm1T were named with the letter T, whereas the remaining 18 samples belonging to clone cmm6-6 were named with the letter S). The 36 samples were then divided into “training data set” and “testing data set”. The size of both data sets was calculated using an option implemented in the MLseq [88]. Twenty-five samples were defined as testing data set and were used by ML to learn and build algorithms from existing data sets, whereas the remaining 11 were defined as testing data set. On this subset, ML makes new predictions and classifies the testing data set in the respective source group. For this purpose, six different ML-based methods classified in: continuous (SVM and SNC), discrete (PLDA, PLDA2, NBLDA) and Voom-based (VoomNSC, VoomDQDA) were individually tested. For each model, the training set accuracy was calculated fractionating the number of correct predictions (true positive + true negative) by the total number (true positive + true negative + false positive + false negative) and using the sparsity values for the proportion of features used in training each model. For example, if sparsity value is similar to zero, less features are used in the classifier. In addition, the same models were also used to predict the miRNAs related to cold stress in testing data set. To achieve the best results, 10-fold cross validation on various combinations of features and classification methods were performed. 

### 8.2. Results

Out of 36 samples analyzed (for further details see Material and methods), 25 were used as training data set, whereas 11 were defined as testing data set. All models showed high accuracy, ranging from 0.89 (SVM) to 0.96 (NSC, PLDA2, VoomNSC), confirming their robustness (Table 1). When we trained each model to predict which group our testing data set belonged to, 11 out of 11 samples were always correctly predicted, with the only exception for SVM, which correctly predicted 10 out of the 11 samples (Table 1). The final aim of the analysis was to find the main features able to discriminate the tolerant from susceptible genotypes as well as to validate previous results. Although all tested methods showed high accuracy, each of them returned a different number of miRNAs as putatively associated with cold tolerance from the 325 selected features. PLDA returned 100 miRNAs, whereas PLDA2 returned 41 and VoomNSC 14. By contrast, SVM, NSC, and VoomDQDA did not return any feature. 

**Figure 4:**
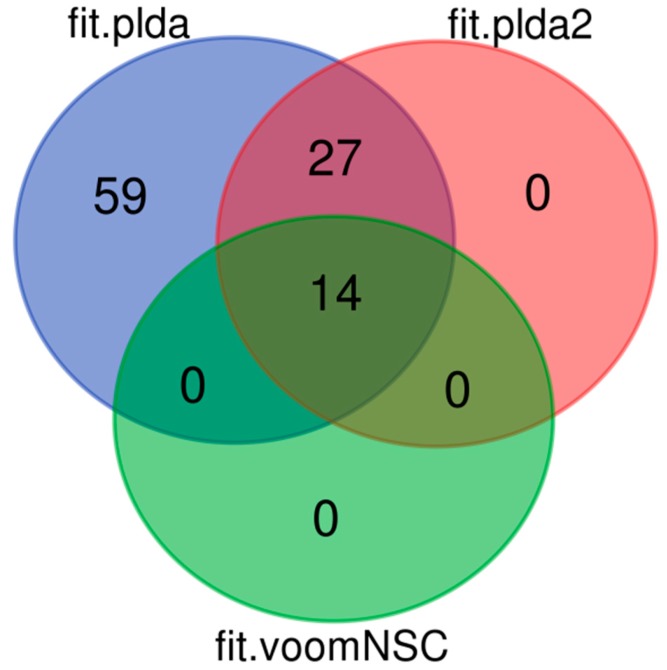
Venn diagram showing the number of common and unique miRNAs associated with cold tolerance predicted by ML models PLDA, PLDA2, and VoomNSC using 325 selected features from published miRNAs data related to cold tolerance in *S. commersonii*.

Among the predicted miRNAs, 14 were in common to all methods and 3 of them had been described as differentially expressed in one of the conditions analyzed in our previous work (Figure 4) [71]. The 3 common miRNAs were all annotated as *S. commersonii*-specific and they regulated a total of 9 different targets. ScMiRNA-6896 regulates the expression of *l-aspartate oxidase*, a pivotal gene implicated in Nicotinamide adenine dinucleotide (NAD^+^) homeostasis in plants. Oono and collaborators [89] revealed that among differentially expressed genes in *Arabidopsis* subjected to cold stress conditions, *aspartate oxidase* (At5g14760) was down-regulated during deacclimation but up-regulated during cold acclimation.

The authors pointed out that although the functions of these genes have not been understood yet, their products may be important for cold acclimation. ScmiRNA-3839 has two targets: an aspartic proteinase-like protein and an endoplasmic reticulum-Golgi intermediate compartment protein. It has been shown in *Arabidopsis* that the overexpression of *aspartic protease* increased abscisic acid sensitivity in guard cells, conferring drought tolerance [90].

This suggests that this target gene may be important also in cold stress response. In addition, aspartic protease *APCB1* was found to be involved in the processing of Bcl-2-Associated Athanogene6 to trigger autophagy and defense mechanisms [91]. MiRNA_5751 had the highest number of targets (six; 2 uncharacterized protein, a serine carboxypeptidase-like 34-like, a hma2 transporter, a pyruvate dehydrogenase e1 component subunit mitochondrial short and membrane-associated progesterone binding protein 4). Among them, pyruvate dehydrogenase e1 component was found suppressed in *Arabidopsis* under drought stress, inducing alteration in bioenergy metabolism [92].

Given the results obtained, it is clear that ML may represent a tool to efficiently and effectively help researchers to overcome the present limitations in smRNAseq projects.

## 9. Conclusions and Future Perspectives

We provided evidence that ML algorithms can be used for classification of miRNAs expression data, allowing researchers to perform classification tasks through a single platform such as MLseq. ML is a rapidly expanding field of research as it is essential for analysis and management of data from different sources, as well for planning and decision strategies. In the future, ML models are expected to be largely applied in the different -omics disciplines, enhancing their integration toward a resolution of key biological questions. This process will require both computational infrastructures and skills in data analysis but also a greater sensitivity and an opened minded approach to novel models to be applied in the various scientific disciplines. This will be facilitated by sharing of knowledge and by interdisciplinary works.

## Figures and Tables

**Table 1:** Prediction of testing data sets. S (susceptible) and R (resistant/tolerant) refer to reference dataset. SVM predict 10 out of 11 samples, being the less accurate model. The training set model accuracy was calculated fractionating the number of correct predictions (true positive + true negative) by the total number (true positive + true negative + false positive + false negative) and using the sparsity values for the proportion of features used in training each model.

Reference Data	*S*	*R*	*S*	*R*	*S*	*S*	*R*	*S*	*S*	*R*	*R*	Model Accuracy
SVM	*S*	*R*	*S*	*R*	*S*	*S*	*R*	*R*	*S*	*R*	*R*	0.89
NSC	*S*	*R*	*S*	*R*	*S*	*S*	*R*	*S*	*S*	*R*	*R*	0.96
PLDA	*S*	*R*	*S*	*R*	*S*	*S*	*R*	*S*	*S*	*R*	*R*	0.93
PLDA2	*S*	*R*	*S*	*R*	*S*	*S*	*R*	*S*	*S*	*R*	*R*	0.96
VoomDLDA	*S*	*R*	*S*	*R*	*S*	*S*	*R*	*S*	*S*	*R*	*R*	0.95
VoomNSC	*S*	*R*	*S*	*R*	*S*	*S*	*R*	*S*	*S*	*R*	*R*	0.96
VoomNBLDA	*S*	*R*	*S*	*R*	*S*	*S*	*R*	*S*	*S*	*R*	*R*	0.95

SVM = Support Vector Machine; NSC = Supervised Normalized Cut; PLDA = Parallel Latent Dirichlet Allocation; Voom = Variance modeling at the observational level; DLDA and NBLDA are diagonal discriminant classifiers.
